# Management of medically unexplained symptoms (MUS): a stepwise integrated model between primary and secondary caremanagement of medically unexplained symptoms (MUS): a stepwise integrated model between primary and secondary care

**DOI:** 10.1192/bjo.2021.73

**Published:** 2021-06-18

**Authors:** Zaineb S Y Al-Dahash, William Loveday, Naomi Law, Mutahira Qureshi, Paul Gallagher, Daniel Turton, Luca Polledri

**Affiliations:** 1 North East London Foundation Trust; 2 East London Foundation Trust; 3 Barts Health NHS Trust

## Abstract

**Aims:**

Description of a model to improve care for patients with Medically Unexplained Symptoms (MUS) by small targeted investment and maximisation of existing resources.

**Background:**

Treatment of MUS presents several challenges including a lack of clarity on the best models of care and limited service provision. Patients typically present with a physical complaint to physical health outlets: here limited confidence in professionals around how to address these often leads to poor patient/doctor experience, inappropriate use of resources and repeated attendance. Evidence shows that integration of care, psychological interventions and upskilling physicians in interventions such as positive communication, can significantly improve outcomes. Psychiatric Liaison Teams (PLT) are positioned at the interface of mental and physical health services and can play a crucial role for these patients, when provided with the right skill-mix.

**Method:**

1FTE Clinical Psychologist specialising in MUS was integrated into the PLT. Pathways to triage between primary, secondary psychology and the new service were agreed, alongside channels of communication and supervision. The job plan included integrated sessions in Gastroenterology, Rheumatology and PLT. The activities included: assessments, formulations and discharges; brief psychological interventions; group sessions for patients; one-day long courses to GP trainees and physicians, and input in specialities MDTs. Clinical outcomes, numbers of patients seen and signposted, teaching sessions and simulation training delivered were collected.

**Result:**

Over 20 months the service was able to process 237 referrals, 35 were managed over the phone. Referral sources: Gastroenterology 32%, Rheumatology 37%, Psychiatric liaison 28%.

116 patients attended 315 face to face appointments and 21 phone contacts were made. Core-10 data show reduction from moderately severe to mild psychological distress in a sample of patients. 58% of patients were referred on for continuing care. The service ran 8 patient groups including sessions on pain management and joint sessions with Rheumatology. It ran 9 one-day long courses for GP and physician trainees, training a total of 120 doctors: feedback showed increased confidence in managing and recognising MUS. Attendances to Emergency Departments covered by Barking Havering and Redbridge and Bart's Health Trusts combined (5 sites) reduced by 22%, saving an estimated £19,200, while ambulance usage in the cohort dropped by 29%, saving an estimated £9072.

**Conclusion:**

The integration of a specialist psychologist with a mix of educational, advisory and clinical role to a PLT can provide an effective and efficient stepped-up model to increase the provision of care for patients with MUS

